# Oxidants, Antioxidants and Thiol Redox Switches in the Control of Regulated Cell Death Pathways

**DOI:** 10.3390/antiox9040309

**Published:** 2020-04-11

**Authors:** Moran Benhar

**Affiliations:** Department of Biochemistry, Rappaport Institute for Research in the Medical Sciences, Faculty of Medicine, Technion-Israel Institute of Technology, Haifa 31096, Israel; benhar@technion.ac.il; Tel.: +972-4-8295376

**Keywords:** cysteine, cell death, apoptosis, necrosis, reactive oxygen species, redox regulation, thioredoxin, glutathione, oxidation, nitrosylation, inflammation, signaling, protein thiols

## Abstract

It is well appreciated that biological reactive oxygen and nitrogen species such as hydrogen peroxide, superoxide and nitric oxide, as well as endogenous antioxidant systems, are important modulators of cell survival and death in diverse organisms and cell types. In addition, oxidative stress, nitrosative stress and dysregulated cell death are implicated in a wide variety of pathological conditions, including cancer, cardiovascular and neurological diseases. Therefore, much effort is devoted to elucidate the molecular mechanisms linking oxidant/antioxidant systems and cell death pathways. This review is focused on thiol redox modifications as a major mechanism by which oxidants and antioxidants influence specific regulated cell death pathways in mammalian cells. Growing evidence indicates that redox modifications of cysteine residues in proteins are involved in the regulation of multiple cell death modalities, including apoptosis, necroptosis and pyroptosis. In addition, recent research suggests that thiol redox switches play a role in the crosstalk between apoptotic and necrotic forms of regulated cell death. Thus, thiol-based redox circuits provide an additional layer of control that determines when and how cells die.

## 1. Introduction

It is now widely recognized that reactive oxygen and nitrogen species (ROS/RNS) play essential roles in many biological processes that span from bacterial growth to mammalian brain function [1,2]. Mammalian cells actively produce ROS and RNS such as superoxide (O_2_^-^), hydrogen peroxide (H_2_O_2_) and nitric oxide (NO) that in turn regulate a broad spectrum of physiological processes, including metabolism, gene transcription/translation, motility, division and differentiation [1,2]. The action of ROS/RNS is counteracted by the activities of endogenous antioxidant systems. The balance between oxidants and antioxidants is thought to be of paramount importance to cellular function, fitness and viability, and its disruption is implicated in some of the most common human disorders, including diabetes, atherosclerosis, cancer, and neurodegenerative diseases [1,2].

For a long time, oxidants have been viewed largely as harmful molecules that cause cell damage and death. From this perspective, antioxidants were considered to act mainly as cellular protectants. On the basis of these perceptions, various models have been put forward, linking redox imbalance to cellular dysfunction and processes of organismal aging and disease. Although these models still serve as useful paradigms, they have undergone significant expansion and refinement over the course of the last three decades. In particular, our understanding of redox regulation of cell survival and death has significantly evolved. Major advances in both the redox and cell death research fields have afforded a detailed view of the cellular roles of oxidant/antioxidant systems as well as of mechanisms of cell death. In turn, these advances have facilitated progress towards elucidating how cell redox and death pathways are interconnected at the molecular level.

An important conceptual advance has been the recognition that ROS/RNS have defined roles in cellular signaling pathways and that pro-/antioxidant systems are tightly regulated and highly compartmentalized [3,4,5]. Indeed, there is extensive evidence that ROS/RNS largely act as signaling molecules, mediating or modulating various cellular responses, among which regulated cell death (RCD) processes are no exception [6,7]. This is particularly well illustrated in the field of apoptosis. Links between ROS/RNS and apoptosis were first revealed in the early 1990s [8,9,10,11,12,13,14], and research in this area has expanded dramatically since then. Reflecting this growth, searching PubMed for the keyword combination “reactive oxygen” and “apoptosis” retrieves ≈35,000 articles. In the last two decades, additional forms of RCD have been discovered and characterized [15,16]. It soon emerged that oxidant and antioxidants are also involved in these non-apoptotic RCD pathways [15,16]. However, for a number of reasons, it has proved challenging to reveal how redox systems and cell death processes are mechanistically connected. In part, the difficulty is related to the diversity and complex biochemistry of ROS and RNS, in particular the ability of ROS/RNS to interact with various cellular targets and impact multiple processes and regulatory circuits that influence cell survival and death. Due to these issues, as well as technical challenges associated with determining the concentrations and localizations of specific oxidants, it is often difficult to establish whether ROS or RNS serve as effector or signaling molecules in cell death responses, or rather, their involvement is secondary to cellular dysfunction and damage.

Despite the various challenges, the past decades have witnessed significant advances in our knowledge of redox control of cell death processes. Accordingly, this review discusses recent progress in our understanding of the roles of oxidants and antioxidants in the control of RCD pathways in mammalian cells. Although thiol oxidants and reductants can impact many cellular constituents and processes that affect cell survival, the present discussion is focused on the roles of redox systems in controlling the molecular machineries that execute specific forms of RCD, in particular, apoptosis, necroptosis, pyroptosis and ferroptosis.

## 2. Reactive Oxygen and Nitrogen Species, Antioxidants, and Thiol-Based Signaling

Mammalian cells continuously generate oxidants as part of normal aerobic metabolism [1,2]. However, oxidant levels fluctuate considerably according to metabolic changes and in response to diverse internal or external cues. Stress stimuli very often affect the cell’s redox systems, resulting in increased levels of oxidants, which in turn can influence the cellular response to these stress insults. Such redox effects are particularly well documented in relation to tumor growth and response to therapy. In this context, it is widely appreciated that antitumor effects elicited by a range of treatment modalities (such as chemotherapy and radiotherapy) frequently involve oxidant-dependent mechanisms [17,18].

ROS and RNS modulate cellular responses by several mechanisms, among which the posttranslational regulation of protein function is thought to constitute a major mechanism of redox control of physiological and pathophysiological processes [19,20,21]. Oxidative protein modifications occur on various amino acid side chains. However, in the context of cell signaling, regulatory oxidative events mainly involve reversible modifications of sulfur- or selenium-containing residues, namely, cysteine, methionine and selenocysteine. Among those, cysteine has received the most attention with regard to redox regulation of cellular function. Cysteine has unique physiochemical properties, which enable it to play important roles in protein function and regulation. Especially, redox modifications of protein cysteine thiols has emerged as a widespread mechanism to control various intracellular pathways triggered or influenced by ROS/RNS [21,22,23,24].

**Figure 1:**
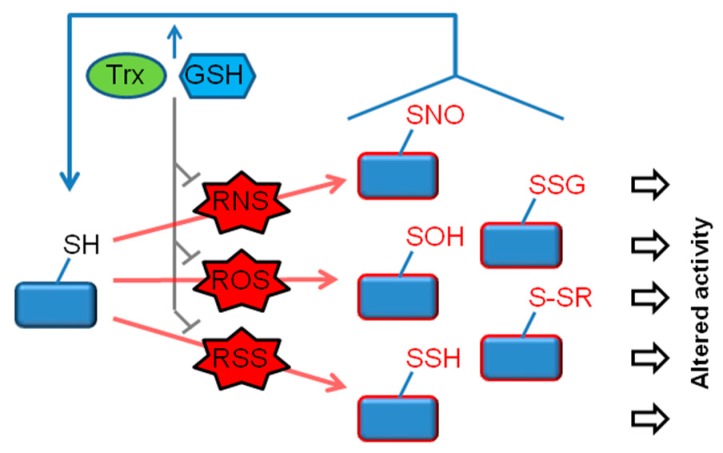
Functional modifications of the thiol proteome. In response to generation of reactive oxygen/nitrogen/sulfur species (ROS/RNS/RSS), protein cysteine thiols (R–SH) undergo a range of oxidative modifications. Of these, major reversible modifications include nitrosylation (R–SNO) (induced by RNS), sulfenylation (R–SOH) (induced by ROS) and persulfidation (R–SSH) (induced by RSS). In some cases, a modification involves a direct reaction between a thiol and an oxidant (e.g. R–SH + H_2_O_2_ → R–SOH), whereas in other cases, multiple reactions are involved. For example, R–SSH can be formed by the reaction of R–SOH with H_2_S. Thiol glutathionylation (R–SSG) and other disulfide forms (R–SSR) are likewise formed by multiple reaction steps. Glutathione (GSH), thioredoxin (Trx) and associated redox enzymes govern the redox state of proteins by two mechanisms, (1) by eliminating ROS/RNS/RSS (e.g., the removal of H_2_O_2_ by GSH- and Trx-dependent peroxidases), and (2) by catalyzing the reduction of oxidized/nitrosylated/persulfidated thiols. Accordingly, the balance between oxidants and GSH/Trx determines the magnitude and duration of each of the redox modifications and thus the downstream functional consequences.

In response to oxidative or nitrosative signals, cysteine thiols undergo a variety of reversible covalent modifications. In particular, RNS such as NO promote the nitrosylation of protein thiols (R–SNO) and certain ROS (such as H_2_O_2_) trigger thiol sulfenylation (R–SOH). Additional reactions of these oxidized thiol groups can lead to thiol glutathionylation (R–SSG) or generate other disulfide species (R–SSR) (Figure 1) [21,22,23,24]. In some situations associated with high ROS levels, thiols may undergo “hyperoxidation”, generating sulfinic (R–SO_2_H) and sulfonic (R–SO_3_H) derivatives (not depicted in the figure). In most cases, sulfinic and sulfonic acids are considered to be irreversible modifications. A notable exception is provided by peroxiredoxin (Prdx) proteins, whereby the sulfinic form of some Prdxs is reduced by the enzyme sulfiredoxin [25]. Another thiol modification that is receiving growing attention is persulfidation (R–SSH), also known as sulfhydration. This modification usually occurs in response to elevation in cellular levels of hydrogen sulfide (H_2_S) or other reactive sulfur species (RSS) [26]. Signaling roles of thiol persulfidation have recently begun to emerge. In addition, recent research points to an important role of persulfidation in protecting proteins from irreversible oxidation [27,28]. The target specificity of the various thiol modifications is an important aspect of redox signaling. A growing number of studies, including comparative proteomic profiling analyses, have revealed a significant degree of specificity among the different redox modifications, supporting the idea that individual modifications have unique rather than overlapping functions [29,30].

The antioxidant protein thioredoxin (Trx), the tripeptide glutathione (GSH), and their system components, are among the main defenses against ROS and RNS in mammalian cells [31,32,33]. In addition, Trx and GSH play a major role in reversing different thiol modifications, including R–SNO, R–SSG and R–SSH [24,33,34]. Trx and GSH systems have both overlapping and non-redundant functions. ROS and RNS catabolism depends on both Trx and GSH, as exemplified by the removal of H_2_O_2_ by GSH- and Trx-dependent peroxidases. Although functional redundancy and crosstalk does exist within the Trx and GSH pathways, proteomic analyses suggest that the two redox systems mostly interact with distinct targets [35]. These observations indicate that Trx and GSH systems control target proteins in a selective rather than a general manner. Thiol antioxidant systems are often upregulated in response to cellular stress; nonetheless, these systems are also vulnerable to excess oxidants and can display decreased or altered activity under conditions of redox stress [25,36]. For example, nitrosylation can inactivate Prdx and Trx reductase proteins [37,38,39]. By governing the magnitude and duration of thiol oxidative modifications on a broad range of cellular proteins, Trx and GSH play a key role in redox-based signaling (Figure 1).

**Figure 2:**
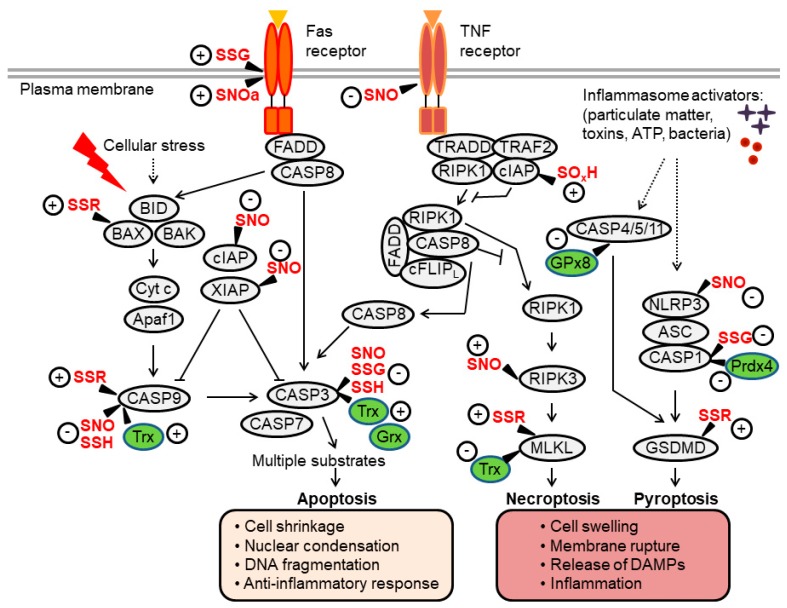
Overview of apoptosis, necroptosis and pyroptosis pathways and their regulation by thiol redox modifications. Extrinsic apoptosis is mediated by the Fas-associated death domain (FADD)-caspase-8 axis, which activates executioner caspases (caspase-3, -7). Intrinsic apoptosis involves mitochondrial outer membrane permeabilization, a process regulated by B-cell lymphoma (BCL)-2-family proteins (such as BID, BAX and BAK). This leads to the formation of the apoptosome (a complex of cytochrome c (Cyt c) and apoptotic protease-activating factor-1 (Apaf-1), which facilitates activation of caspase-9 upstream of caspase-3/7. Activation of the tumor necrosis factor (TNF) receptor can induce apoptosis through a signaling cascade that activates caspase-8. Under conditions of caspase-8 inhibition, a protein complex consisting of receptor-interacting kinase (RIPK)1/3 and mixed lineage kinase-like (MLKL) is formed, promoting the execution of necroptotic cell death. In inflammatory cells such as macrophages, pathogens and danger signals promote the assembly of inflammasome complexes, which activate caspase-1 or, in some settings, mouse caspase-11 (human caspase-4/-5). These inflammatory caspases cleave gasdermin D (GSDMD) to induce pyroptosis. Proteins directly regulated by thiol modifications or redox enzymes (thioredoxin [Trx], glutaredoxin [Grx], glutathione peroxidase [GPx]) are highlighted, where “+” and “-“ indicate positive and negative effects on protein activity, respectively (see the main text for further details). Through these thiol redox switches, oxidants and Trx/GSH systems influence regulated cell death (RCD) processes and the crosstalk between them. SNO, nitrosylation; SOH, sulfenylation; SSG, glutathionylation; SSR, disulfide formation; SO_x_H, oxidation or hyperoxidation.

Accumulating research shows that oxidative thiol modifications, governed by Trx and GSH, regulate multiple proteins that play critical roles in cell survival and death. These “thiol redox switches” thereby modulate the activation of several RCD pathways, including apoptosis, necroptosis and pyroptosis (schematically summarized in Figure 2). The following sections discuss the roles and mechanisms by which thiol modifications influence specific modes of RCD.

## 3. Thiol Redox Control of Apoptosis

Apoptosis is the most studied and best characterized form of RCD. It is activated via two distinct pathways, referred to as the intrinsic and the extrinsic apoptotic pathways (Figure 2) [40,41]. As detailed below, these caspase-activating pathways act as signaling modules that integrate various death-inducing signals.

The intrinsic apoptotic pathway is activated by various stressors that are capable of inducing mitochondrial outer membrane permeabilization (MOMP), a process driven by proapoptotic members of the B-cell lymphoma-2 (BCL-2) family of proteins, especially BCL-2 associated X, apoptosis regulator (BAX) and BCL-2 antagonist/killer 1 (BAK). MOMP leads to the release of multiple proteins from the mitochondrial intermembrane space to drive apoptosis. In particular, the release of cytochrome c (Cyt c) triggers the rapid oligomerization of apoptotic protease-activating factor-1 (Apaf-1), which recruits caspase-9 into the apoptosome. Active caspase-9 then directly cleaves and activates downstream effector caspases (such as caspase-3), resulting in apoptosis. The extrinsic apoptosis pathway is activated from outside the cell by proapoptotic ligands that interact with specialized cell surface death receptors such as tumor necrosis factor (TNF) receptor (TNFR) or Fas (also known as CD95 or APO-1). Upon activation, the Fas receptor forms the death-inducing signaling complex (DISC) by recruiting the adapter Fas-associated death domain (FADD) and procaspases-8 (or -10), resulting in caspase activation. Caspase-8/-10 then activate effector caspases to execute apoptosis. Through a related (but distinct) mechanism, TNFR can also engage the caspase-8/-3 cascade (Figure 2) [40,41].

Oxidants and antioxidants regulate apoptosis through multiple mechanisms, in particular via modifications of proteins and lipids. With regard to lipids, an important example is the oxidation of cardiolipin in the mitochondria, which can be triggered by ROS or by the cardiolipin–Cyt c complex. Because oxidized cardiolipin has a much lower affinity for Cyt c than the non-oxidized form, its oxidation promotes Cyt c release from mitochondria and propagation of the apoptotic signal [42,43]. 

The next sections will focus on the redox regulation of protein components of the apoptotic machinery. As will be discussed below, multiple apoptotic proteins are regulated by thiol redox modifications. These modifications can exert both positive and negative effects on the apoptotic process.

### 3.1. Apoptotic Caspases

#### 3.1.1. Caspase-3 Regulation by S-Nitrosylation and Oxidation

Caspases are cysteine proteases with main specificity for aspartic acid (Asp) residues, cleaving their substrates after tetrapeptide sequences containing Asp in the P1 position [44]. Caspases are synthesized as inactive single chain zymogens (procaspases), and they are obligate heterodimers in their active forms. Caspase zymogens consist of an N-terminal prodomain and a C-terminal protease domain, which has a large and a small subunit that contains the catalytic cysteine residue [44]. The catalytic cysteines of several caspases bear highly nucleophilic thiols, which readily react with oxidants. Indeed, early studies have reported that multiple caspases are rapidly (yet reversibly) inactivated upon exposure to NO donors or H_2_O_2_ [45,46,47,48]. These early studies provided initial insights into redox regulation of cell death via caspase oxidation, and advanced an understanding of the dual role of oxidants, being able to activate or inhibit apoptosis [48]. However, the physiological relevance of these findings was not clear for some time. In the late 1990s, several studies provided evidence that S-nitrosylation serves as a physiological mechanism to suppress caspase-mediated apoptosis, providing a molecular explanation for the antiapoptotic action of NO [49,50,51,52]. In this regard, the redox regulation of caspase-3 has been mostly characterized. Studies in lymphocytes have revealed that cytosolic procaspase-3 is non-nitrosylated under basal steady-state conditions, whereas mitochondria-associated procaspase-3 is constitutively nitrosylated [52,53]. It was further discovered that certain apoptotic stimuli such as Fas ligand trigger the denitrosylation and activation of mitochondrial procaspase-3, and that Trx proteins mediate both basal and stimulus-induced denitrosylation [53,54]. In particular, it emerged that the mitochondrial Trx/Trx reductase system (Trx2/TrxR2) plays a critical role in this process [55]. Indeed, Trx2-mediated denitrosylation of caspase-3 was found to regulate death receptor-mediated apoptosis in lymphocytes [54], melanoma cells [56], and cells of the hippocampus [57]. Distinct from these findings, it was reported that under some conditions, the opposite nitroso transfer–from Trx to procaspase-3–can occur, leading to decreased apoptosis [58]. Additionally, a recent study showed that reduced Trx can inhibit caspase-6 activity, whereas oxidized Trx has the opposite effect; however, the exact underlying mechanism requires further elucidation [59].

Recent intriguing evidence suggests that nitrosylation of caspase-3 regulates microglia activation in the context of glioma expansion. Specifically, suppression of basal caspase-3 activity in microglia was found to be associated with microglia polarization toward a tumor-supportive phenotype [60]. It was revealed that glioma-induced microglia conversion is coupled to increased nitrosylation of mitochondria-associated caspase-3 through inhibition of Trx2. The findings suggest a model in which nitrosylation of mitochondrial caspase-3 regulates microglial tumor-supporting function [60]. This work highlights the importance of thiol redox control in regulating a non-apoptotic function of a caspase.

#### 3.1.2. Caspase-3 Regulation by S-Glutathionylation

Several studies provided evidence for caspase regulation via glutathionylation [61,62]. In particular, it was reported that procaspase-3 is basally glutathionylated in endothelial cells. Exposure to TNF promotes glutaredoxin (Grx)-mediated deglutathionylation of procaspase-3, a process that positively regulates apoptosis [61]. Of note, mutagenesis experiments suggested that two non-catalytic thiols of caspase-3 (Cys-184 and Cys-220) mediate these effects [61]. However, the way in which glutathionylation of these residues inhibits caspase activity has not been elucidated.

#### 3.1.3. Caspase-3/9 Regulation by S-Persulfidation

New evidence shows that thiol persulfidation regulates the activity of caspase-3 and -9 [63]. Specifically, exposure of HeLa cancer cells to polysulfides triggers the persulfidation and deactivation of cleaved caspase-3 and -9. The Trx/TrxR system mediates caspase depersulfidation, a process required for protease activation. Of particular interest, the new findings show that procaspase-3 and -9 are constitutively persulfidated in resting HeLa cells and undergo Trx/TrxR-dependent depersulfidation during their processing and activation in response to an apoptotic stimulus [63]. These observations support the idea that persulfidation serves as a safeguard mechanism in apoptosis, whereby Trx-catalyzed persulfide removal is necessary to enable caspase activation and propagation of the apoptotic signal. This study also revealed a higher turnover of persulfides on cleaved caspases as compared to the proenzymes. This finding may be explained by the conformational changes that occur during the proteolytic maturation of caspases, which render the catalytic thiol more accessible to the solvent [64].

#### 3.1.4. Caspase-9 Regulation by S-Nitrosylation and Oxidation

Similar to caspase-3, initiator caspase-9 is also subject to regulation by S-nitrosylation. It was shown that nitrosylation inhibits caspase-9 activity and its denitrosylation promotes cancer cell apoptosis [65,66]. Conversely, other studies have found that oxidative modifications can also promote, rather than inhibit, the activation of caspase-9. One study proposed a model whereby an oxidant signal triggers the rapid activation of caspase-9 in mitochondria via disulfide-mediated dimerization [67]. Another study also suggested that oxidation of caspase-9 facilitates its activation, however, a different mechanism was implicated [68]. Specifically, oxidants were reported to promote a disulfide-mediated interaction between caspase-9 with Apaf-1. Mutagenesis analysis indicated that Cys-403 of caspase-9 mediates this interaction [68]. It is important to note that these studies involved cell exposure to exogenous H_2_O_2_ or to the thiol oxidant diamide; as such, the significance of thiol oxidation in physiological caspase-9 regulation remains to be further investigated.

#### 3.1.5. The Pro- versus Anti-Apoptotic Effects of Redoxins

As indicated above, the Trx system can promote apoptotic signaling by reversing nitrosylation or persulfidation of apoptotic caspases. Likewise, the deglutathionylating enzyme Grx potentiates caspase-3 signaling. These apoptosis-promoting effects of Trx and Grx may appear counterintuitive, considering the many studies emphasizing the cytoprotective and antiapoptotic functions of these ubiquitous redoxins. The antiapoptotic effects of Trx and Grx are mediated in part through their interactions with a number of signaling proteins, such as apoptosis signal-regulating kinase 1 (ASK1) (reviewed in [69,70]). Nevertheless, it was recognized long ago that Trx can also mediate proapoptotic effects [71,72]. Along similar lines, GSH is commonly associated with pro-survival effects, yet is required for caspase activation in some settings [73]. These observations highlight the complex role of thiol reducing systems in apoptosis regulation. As discussed below, the consideration of redox control across multiple RCD modalities may clarify some of these apparent paradoxes. 

### 3.2. BAX

As noted above, activation of the intrinsic apoptosis pathway involves the induction of MOMP, a process regulated by proteins of the BCL-2 family [40,41]. BCL-2 proteins are characterized by the presence of one to four BCL-2 homology (BH) domains. The proapoptotic family members include a BAX-like subfamily (BAX, BAK, and BOK), as well as a “BH3-only” subfamily (BIM, BID, PUMA, BAD, NOXA). Activated BAX and BAK proteins stimulate MOMP and the release of Cyt c by forming pores in the outer mitochondrial membrane (OMM). Antiapoptotic family members (such as BCL-2 and BCL-XL) can neutralize BAX-like family members, thereby preventing BAX/BAK-mediated MOMP.

In healthy cells, BAX localizes to the cytoplasm and translocates to the mitochondria in response to an apoptotic trigger. In some cells, exposure to H_2_O_2_ can promote the mitochondrial translocation of BAX that is accompanied by disulfide dimerization, implying that BAX oxidation is an early regulatory event in apoptosis [74]. Studies in human colon adenocarcinoma cells indicate that H_2_O_2_ can trigger a conformational change in BAX, promoting its translocation and oligomerization in the OMM. H_2_O_2_-induced BAX activation reportedly depends on a conserved cysteine residue (Cys-62), which is located in the BH3 domain [75]; however, how oxidation affects BAX conformation is still vague. Overall, these studies support a role for oxidative activation of BAX is some settings of apoptosis, in particular, those involving significant ROS generation.

### 3.3. XIAP and cIAP 

The activation of several apoptotic caspases is counteracted by a family of proteins knows as inhibitors of apoptosis (IAPs). This family consist of structurally distinct proteins, including X-linked (XIAP), cellular (cIAP1, cIAP2), neuronal (NIAP) and survivin, among others. These IAPs play pivotal roles in apoptosis control, development, homeostasis, and disease [76,77]. Structurally, IAPs are approximately 70 amino acids long and contain zinc finger baculovirus IAP repeat (BIR) domains that are responsible for the inhibitory properties of IAPs, by preventing the conversion of zymogenic procaspases to active caspases. XIAP is one of the major IAPs, being able to bind and inhibit caspases-9 and -3. In addition, XIAP can ubiquitinate binding partners and thereby may permanently inactivate caspases or promote their degradation. 

Two independent studies reported that S-nitrosylation modulates XIAP activity [78,79]. Both investigations found that S-nitrosylation compromises XIAP activity, resulting in increased apoptosis. It was proposed that elevated nitrosylation of XIAP may contribute to neurodegeneration [78,79]. It should be noted, however, that the two studies differed regarding the site of the modification and the mechanism underlying enzyme inhibition. Whereas one study reported that nitrosylation mostly perturbs binding of XIAP to caspase-3 [78], the other study suggested that the modification impairs its E3 ligase activity [79]. As such, the exact effect of nitrosylation on XIAP activity remains to be fully elucidated.

Oxidation of cIAP2 was recently shown to regulate TNF-induced apoptosis in cancer cells [80]. This discovery emerged from observations related to the apoptosis-modulating effects of Prdx-1 and 2. It was revealed that these two closely-related peroxidases act by distinct mechanisms, whereby Prdx-1 influences the DNA damage response while Prdx-2 regulates the activity of receptor-interacting kinase 1 (RIP1, also known as RIPK1), a kinase important in transmitting apoptotic signaling downstream of the TNFR1 (Figure 2). It was demonstrated that loss of Prdx-2 selectively switches the canonical apoptosis pathway to the RIPK1-dependent pathway by inducing cIAP depletion. In healthy cells, Prdx-2 interacts with and preserves the stability of cIAP in caveolar membrane microdomains. In stimulated cells, Prdx-2 deficiency enhances H_2_O_2_-mediated hyperoxidation of Cys-308 residue in the BIR3 domain of cIAP, thereby triggering the dimerization and activation of cIAP E3 ligase. Consequently, cIAP undergoes degradation, unleashing RIPK1 activity and apoptosis [80]. How oxidation of Cys-308 leads to ligand-independent dimerization and activation of cIAP E3 ligase is currently unclear and warrants further investigation.

Another recent report shows that cancer cell stimulation with the NO donor glyceryl trinitrate (GTN) promotes TNF-mediated cell death in colon and mammary cancer cells [81]. Mechanistically, GTN induces the nitrosylation of cIAP1 on two cysteines residues (Cys-571 and Cys-574) within the RING domain, which leads to inhibition of its E3 ubiquitin ligase activity towards RIPK1. In turn, decreased ubiquitination of RIPK1 results in its stabilization, thereby enhancing apoptotic signaling [81]. Whether endogenous NO can similarly regulate cIAP1 function is currently unknown. 

### 3.4. Death Receptors

Studies by Janssen-Heininger and colleagues have provided evidence for redox regulation of the death receptor Fas [82]. The findings show that cell stimulation with Fas ligand induces S-glutathionylation of Fas at Cys-294. This modification, which occurs after caspase-dependent degradation of Grx1, potentiates caspase activation and apoptosis [82]. In a subsequent study, the same group provided further mechanistic insights into Fas regulation by thiol redox. It was shown that a pool of latent Fas in the endoplasmic reticulum (ER) is selectively oxidized during apoptosis by a mechanism that involves ER-localized ERp57 and glutathione S-transferase π (GSTP), a protein disulfide isomerase and a catalyst of S-glutathionylation, respectively. These stimulus-induced redox events result in elevated surface Fas, which in turn promotes DISC assembly, caspase activation, and cell death [83].

Nitrosylation of death receptors has been reported in several studies. In one study, treatment of colon and mammary cancer cells with GTN induced S-nitrosylation of Fas [84]. The modification targets cysteine residues 199 and 304 in the cytoplasmic part of Fas. The findings suggest that nitrosylation promotes recruitment of the receptor to lipid rafts, thus sensitizing the cells to apoptosis [84]. Another NO donor, nitrosylcobalamin (NO-Cbl) is known to induce apoptosis in several types of cancer cells via the extrinsic apoptotic pathway. Mechanistic studies suggest that NO-Cbl triggers the nitrosylation of death receptor DR4 (TRAILR) [85]. How nitrosylation modulates receptor activity is not known, but it was speculated that it may cause a conformational change and render the receptor more sensitive to activation by its ligand [85]. Distinct from these findings, a recent study indicates that decreased nitrosylation promotes TNF-mediated apoptosis in hepatoblastoma cells [86]. Specifically, apoptosis elicited by the multi-kinase inhibitor sorafenib is associated with a decrease in nitrosylation of TNFR1. The data support a model in which the downregulation of receptor nitrosylation by sorafenib may shift the balance from survival to apoptosis [86].

### 3.5. Other Apoptosis Regulators

Studies by Rojanasakul and colleagues suggest that additional components of the apoptotic machinery could be regulated by S-nitrosylation. In particular, nitrosylation of BCL-2 and FADD-like apoptosis regulator (CFLAR; best known as c-FLIP, a protein that blocks recruitment and processing of caspase-8 at the DISC) was reported to inhibit their degradation and thereby exert an antiapoptotic effect (reviewed in [87]).

## 4. Thiol Redox Control of Necroptosis

Discoveries made in the last two decades revealed the existence of several forms of regulated necrosis pathways, including necroptosis, pyroptosis, and ferroptosis [15,16]. These advances have transformed the field of cell death by demonstrating that cell necrosis is not merely accidental, but rather, can be a regulated process, mediated by a genetically encoded machinery. Importantly, the specific mode by which cells die has functional consequences to the organism. This is probably best exemplified in the immune-inflammatory response, in which apoptosis is generally considered as an immunologically-silent event, whereas necrotic cell death usually elicits a potent pro-inflammatory response [15,16]. 

Necroptosis is a form of RCD that is typically activated by specific death receptors such as TNFR1 and pathogen recognition receptors such as toll-like receptor (TLR)-3 and TLR-4 [88]. Activation of these receptors triggers the formation of a RIPK1-RIPK3 cell death platform, termed the necrosome, followed by RIPK3-mediated phosphorylation of mixed lineage kinase-like (MLKL), which triggers MLKL oligomerization and membrane association, resulting in plasma membrane damage and cell death (Figure 2). Because RIPK1 and 3 have several non-necroptotic functions, mammalian necroptosis is best defined by its requirement for MLKL. Importantly, activation of necroptosis usually requires the inactivation of apoptotic signaling components, in particular, of specific IAP proteins (cIAP1/2) and caspase-8, which ubiquitinate or cleave RIPK1/3, respectively, in order to limit necroptosis [15,16]. Recent findings have implicated ROS in necroptosis regulation. Evidence for the involvement of thiol redox control in necroptotic cell death is next discussed.

### 4.1. RIPK1/3

Although NO has been extensively studied in relation to apoptosis, very little is known about its role in necroptosis. One study provided evidence that nitrosylation of RIPK3 is involved in ischemic neuronal injury by activating necroptosis [89]. The observations suggest that ischemia provokes NO synthase-dependent nitrosylation of RIPK3 in a manner that promotes its phosphorylation and activity [89]. However, the molecular link between elevated RIPK3 nitrosylation and phosphorylation remains to be identified. 

A body of evidence suggests that mitochondrial ROS promote necroptosis, but the underlying mechanisms are only beginning to be understood. Recent research has revealed that three cysteines in RIPK1 are required for sensing ROS during necroptotic cell death [90]. Oxidation of the three cysteines leads to the formation of disulfide bond-linked high molecular weight aggregate of RIPK1, resulting in increased autophosphorylation that facilitates necrosome formation [90].

### 4.2. MLKL

A recent report by Wang and colleagues showed that MLKL forms disulfide bond-dependent large polymers during necroptosis [91]. These polymers are detected in both human and mouse cells undergoing necroptosis and are independent of RIPK1/RIPK3 fibers. Importantly, MLKL mutants that cannot form polymers also fail to efficiently induce necroptosis. These results indicate that disulfide bond-dependent, amyloid-like MLKL polymers are necessary to induce necroptosis [91]. In a subsequent study, the same group showed that recombinant Trx1 preferentially binds to monomeric MLKL and blocks MLKL disulfide bond formation and polymerization *in vitro*. Furthermore, knockdown of endogenous Trx1 promotes MLKL polymerization and sensitizes cells to necroptosis [92]. These findings point to Trx1 as a negative regulator of necroptosis by virtue of maintaining MLKL in a reduced, inactive state. In addition, Trx1 may also inhibit necroptotic signaling upstream of MLKL by interfering with the formation of necrosome-like complexes [93].

## 5. Thiol Redox Control of Pyroptosis

Pyroptosis is a form of necrotic RCD that has been mostly studied in innate immune cells [16]. It is often defined as caspase-1-mediated cell death, although recent studies have revealed that additional caspases are capable of triggering pyroptosis. Activation of caspase-1 (and other inflammatory caspases) typically occurs within inflammasome complexes. The execution of pyroptotic cell death is a result of caspase-mediated cleavage and activation of proteins that belong to the pore-forming gasdermin gene family [94]. To date, caspases-1, -4, -5, and -11 have been shown to target gasdermin D (GSDMD), whereas caspase-3 can process gasdermin E (GSDME/DFNA5). The cleavage of gasdermin releases an activated N-terminal region that binds to acidic phospholipids, such as phosphoinositides found on the inner leaflet of the mammalian plasma membrane, to generate oligomeric death-inducing pores. Thus, pyroptosis can be defined as a form of RCD that critically depends on the formation of plasma membrane pores by members of the gasdermin protein family, often as a consequence of inflammatory caspase activation [94]. As detailed below, oxidative thiol modifications of gasdermins, caspases or inflammasome sensors can influence the pyroptotic process.

### 5.1. Inflammasome-Caspase-1 Pathway

Inflammasomes are molecular platforms activated upon cellular infection or stress that trigger the maturation of proinflammatory cytokines such as interleukin (IL)-1β, as well as promoting the induction of pyroptosis, two processes that play a key role in innate immune defenses [95]. The canonical inflammasomes are composed of at least three components: an inflammatory caspase, such as caspase-1, an adapter molecule, such as apoptosis speck protein (ASC), and a sensor protein, such as nucleotide-binding domain (NOD)-like receptor protein 3 (NLRP3). Many studies support the notion that ROS are involved in the activation of several inflammasomes, in particular, the NLRP3 inflammasome; however, how ROS affect inflammasome activation is still a matter of debate and ongoing investigation [96]. In this regard, some evidence suggests that the Trx inhibitor protein Txnip links ROS to inflammasome activation. Mechanistically, an increase in ROS can lead to the dissociation of Txnip from oxidized Trx1, followed by Txnip association with and activation of NLRP3 (reviewed in [97]). Nonetheless, the role of Txnip in inflammasome regulation remains controversial. A very recent study supports the idea that the Trx system positively regulates NLRP3 activation independently of Txnip [98].

Accumulating evidence indicates that thiol oxidation is involved in inflammasome regulation. An early study showed that in superoxide dismutase 1 (SOD1)-deficient macrophages, higher superoxide production specifically inhibits caspase-1 via thiol glutathionylation of cysteine residues Cys-397 and Cys-362 [99]. Consistently, SOD1-deficient mice produce less caspase-1-dependent cytokines and are less susceptible to lipopolysaccharide-induced septic shock [99]. Recently, O’Neill and colleagues reported that the protein NEK7, which controls NLRP3 inflammasome activation, is also regulated by glutathionylation. It was shown that glutathione transferase omega 1-1 mediates the deglutathionylation of NEK7, thereby promoting inflammasome activation [100]. 

Two very recent studies have revealed a new twist on the mechanism by which thiol redox regulates pyroptosis. The first study uncovered a link between caspase-1 and the thiol peroxidase Prdx-4, by showing that Prdx-4 negatively regulates IL-1β generation and cell death through inflammasome modulation [101]. Specifically, Prdx-4 forms a redox-sensitive regulatory complex with caspase-1 via Cys-397 that leads to caspase-1 sequestration and inactivation. Interestingly, the interaction between Prdx-4 and caspase-1 takes place in extracellular vesicles, implying its involvement in inflammasome-mediated propagation of inflammatory signals between cells [101]. In the second study, glutathione peroxidase (GPx)-8 was shown to inhibit the activation of another pyroptosis-related protease, caspase-4/11 [102]. GPx8 binds covalently to caspase-4/11 through a disulfide linkage between Cys-79 of GPx8 and Cys-118 of caspase-4, thereby attenuating caspase-4/11 oligomerization and activation. This mechanism may account for the ability of GPx8 to protect against colitis [102].

Studies in the past decade have shown that S-nitrosylation negatively regulates the NLRP3 inflammasome [103,104]. The available evidence supports the idea that nitrosylation of NLRP3, rather than caspase-1, mediates the inhibitory effect. This mechanism may underlie, at least in part, the anti-inflammatory action of NO (independent of its anti-microbial function), which is important in the host defense against infectious agents [105].

Along similar lines, various studies have demonstrated the anti-inflammatory effects of H_2_S [26]. Recently, H_2_S was reported to antagonize inflammasome activation by interfering with steps in the activation of caspase-1 [106]. However, additional investigation is needed in order to reveal the exact molecular mechanism by which H_2_S regulates inflammasome activation, and to assess the possible involvement of thiol persulfidation in this effect.

### 5.2. Gasdermin-D

A very recent study suggests that oxidation of GSDMD plays an important role in regulating pyroptosis [107]. The findings support a model in which mitochondrial ROS trigger the oxidation of GSDMD on four cysteine residues, promoting its cleavage by caspase-1 and enhancing pyroptotic cell death [107]. Although further structural and mechanistic details remain to be uncovered, these observations provide a novel link between thiol oxidation and pyroptosis. 

## 6. Redox Control of Additional Modes of RCD

Beyond the mechanisms discussed above, RCD pathways encompasses additional cell death modalities, all of which are considered to be regulated, inasmuch as specific genetic or pharmacological manipulations are able to interrupt the lethal cascade [108]. 

### 6.1. Mitochondrial Permeability Transition–Mediated Necrosis

Mitochondrial permeability transition (MPT)-initiated necrosis is a form of RCD triggered by severe oxidative stress and cytosolic Ca^2+^ overload [16,108]. MPT involves the abrupt loss of the impermeability of the inner mitochondrial membrane (IMM) to small solutes, resulting in rapid dissipation of transmembrane potential (Δψm), osmotic breakdown of both mitochondrial membranes, leading to cell death. At the molecular level, MPT-driven necrosis entails the opening of the so-called “permeability transition pore complex” (PTPC), a supramolecular complex assembled at the junctions between the IMM and OMM. The composition and mode of action of this complex is a topic of much debate and ongoing research. Reversible oxidative modifications of components of PTPC, such as cyclophilin D, could be involved in the progression of MPT-mediated necrotic cell death but the exact mechanisms remain to be fully elucidated [109,110,111]. 

### 6.2. Ferroptosis

**Figure 3:**
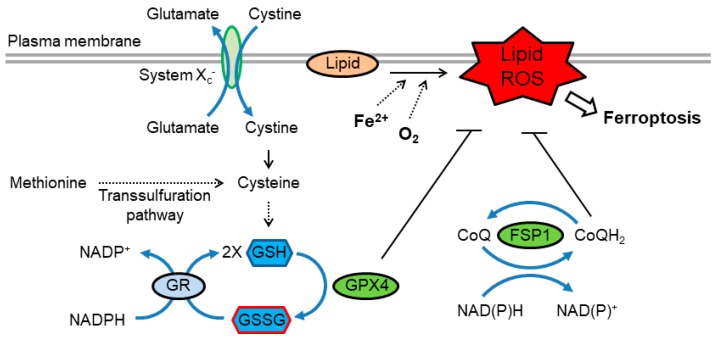
Redox regulation of ferroptosis. Ferroptosis is driven by lipid peroxidation, which is catalyzed by redox-active iron. Cysteine availability, which is regulated by cystine uptake via system x_c_^-^ or through the transsulfuration pathway, is essential for efficient synthesis of GSH. GPX4 utilizes GSH to reduce lipid hydroperoxides, thereby suppressing ferroptosis. GSSG is reduced to GSH by GR using NADPH. In addition, reduced coenzyme Q (CoQH_2_) acts as a lipophilic radical-trapping antioxidant that halts the propagation of lipid peroxidation. The CoQ oxidoreductase ferroptosis suppressor protein 1 (FSP1) maintains CoQH_2_ levels and acts parallel to GPX4 to inhibit ferroptosis. FSP1, ferroptosis suppressor protein 1; GSH, glutathione; GPX4, glutathione peroxidase 4; GSSG, glutathione disulfide; GR, glutathione reductase.

Ferroptosis is a form of RCD characterized by the iron-dependent accumulation of toxic levels of lipid hydroperoxides [112,113]. Ferroptosis is often triggered under conditions where intracellular levels of GSH significantly drop. This leads to a decrease in the activity of the GSH-dependent peroxidase GPX4. This selenoprotein is a major enzyme that reduces lipid peroxides, and therefore, its deactivation constitutes an important event is the progression of ferroptosis [112,113] (Figure 3). It was demonstrated that selenocysteine-based GPX4 catalysis plays a key role in protection against ferroptosis, at least in some cell types [114]. Thus, cellular pathways that regulate GSH levels or selenoprotein biosynthesis modulate ferroptosis. In particular, because cysteine is a precursor of GSH, fluctuations in the intracellular level of cysteine profoundly affect sensitivity to ferroptosis. Endogenous cysteine levels are mostly determined by (1) uptake of extracellular cystine by cystine/glutamate antiporter known as system x_c_^-^ and the subsequent reduction of cystine to cysteine, and (2) the transsulfuration pathway, in which methionine is used as a sulfur donor and is converted to cysteine through the intermediates homocysteine and cystathionine. Accordingly, modulation of either of these pathways impacts upon ferroptosis [112,113]. It is worth pointing out that loss of intracellular GSH has also been implicated in apoptosis. In fact, GSH efflux frequently occurs during early stages of apoptosis [115]. It is currently not well defined how GSH loss is coupled to the apoptotic machinery, and what additional factors determine the cellular decision to engage apoptosis *versus* ferroptosis. 

Recent studies have uncovered additional redox-related pathways that regulate ferroptosis. It was found that the flavoprotein apoptosis-inducing factor mitochondria-associated 2 (AIFM2) acts as an anti-ferroptotic factor [116,117]. AIFM2, has, therefore, been renamed ferroptosis suppressor protein 1 (FSP1). Suppression of ferroptosis by FSP1 is mediated by ubiquinone (coenzyme Q, also known as CoQ or CoQ_10_ in humans), whereby the reduced form, ubiquinol (CoQH_2_), traps lipid peroxyl radicals that mediate lipid peroxidation. FSP1 catalyzes the regeneration of CoQH_2_ using NAD(P)H [116,117] (Figure 3).

Given the crosstalk between the Trx and GSH pathways [33], it seem likely that the Trx system will play a role in ferroptosis in some situations. This notion is supported by a recent study that identified a novel small molecule, named “ferroptocide”, which induces ferroptotic death of cancer cells [118]. Further mechanistic studies including target identification revealed that ferroptocide covalently modifies Trx, indicating that Trx may act as a suppressor of ferroptosis in some settings [118].

Lastly, it is worth mentioning that NO is a potent inhibitor of lipid peroxidation, and therefore, it is expected to inhibit ferroptosis. Indeed, the antiferroptotic action of NO has recently been demonstrated [119].

### 6.3. Parthanatos

Parthanatos is a form of RCD mediated by the hyperactivation of poly(ADP-ribose) polymerase 1 (PARP1) [120]. Activation of parthanatos can be triggered in response to oxidative/nitrosative stress, DNA damage, and other insults. PARP1 hyperactivation mediates cytotoxic effects by causing depletion of NAD^+^ and ATP, which results in a bioenergetic and redox collapse, as well as by promoting the accumulation of poly(ADP-ribose) polymers and poly(ADP-ribosyl)ated proteins at mitochondria, leading to Δψm dissipation and MOMP. One of the key steps of parthanatos is the binding of poly(ADP-ribose) polymers to apoptosis-inducing factor mitochondria associated 1 (AIFM1; also known as AIF). This results in the release of AIF into the cytosol and its translocation into the nucleus, where it promotes large-scale DNA fragmentation and chromatin condensation [120]. Although ROS and RNS are associated with parthanatos, at present, it is unclear whether thiol oxidation or nitrosylation is involved in this RCD pathway.

### 6.4. Oxeiptosis

Oxeiptosis is a recently described signaling pathway that couples ROS to a non-canonical cell death pathway that involves Kelch ECH associating protein 1 (Keap1) [121]. It is well known that in non-stressed cells, Keap1 inhibits the activity of the transcription factor nuclear factor erythroid 2-related factor 2 (Nrf2). Oxidative/nitrosative insults lead to oxidation of Keap1 and release of Nrf2, which then translocates to the nucleus to activate transcription of cytoprotective genes. Oxeiptosis involves another interaction of Keap1, with a mitochondrial serine-threonine phosphatase called PGAM5. ROS-dependent oxidation of Keap1 leads to its dissociation from PGAM5, which then can dephosphorylate and activate the protein AIFM1, resulting in cell death [121]. 

## 7. Thiol Redox and the Apoptosis-to-Necrosis Switch 

**Figure 4:**
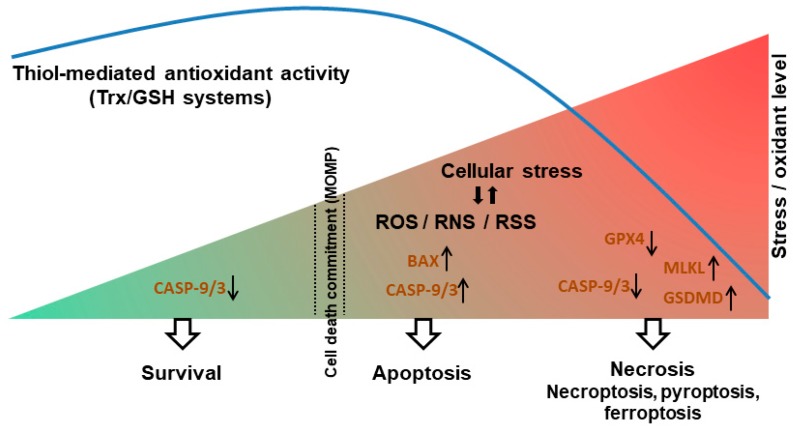
A model of the redox-dependent switch between apoptosis and necrosis. In non-stressed cells, thiol antioxidants maintain low levels of oxidants (ROS/RNS/RSS). Upon stress, elevated levels of oxidants promote the induction of apoptosis by stimulating the BAX→Cyt c→caspase-9→ caspase-3 cascade. The activity of Trx/GSH systems is required to enable caspase-9/3 activation and propagation of the apoptotic signal (a redox checkpoint). Under conditions of excess levels of oxidants and/or compromised antioxidant activity, thiol oxidation of caspase-9/3 limits apoptosis, whereas oxidation of MLKL and GSDMD promotes necroptosis and pyroptosis respectively. Low GSH levels also lead to decreased GPX4 activity, promoting ferroptosis. Upward and downward black arrows indicate increased or decreased protein activity, respectively.

It is becoming clear that there is extensive crosstalk between RCD modalities. As mentioned above, negative regulation of necroptosis by some apoptotic caspases is well established, and other mechanisms of cross-regulation between apoptosis, necroptosis and pyroptosis are being revealed [108,122]. The discoveries herein described support the idea that thiol redox switches play an active role in RCD-related crosstalk. In particular, the findings suggests that oxidants and Trx/GSH coordinately control critical components of RCD pathways and thereby govern the propensity of cells to undergo apoptosis *versus* necrosis. Accordingly, one can propose the following model of RCD control by “redox checkpoints” as detailed below and illustrated in Figure 4. 

According to the proposed model, in response to a stress stimulus, an oxidant burst is needed in order to reach a “death threshold” (such as MOMP) and activate apoptosis. The oxidant signal could be mediated via oxidation of BAX or cardiolipin, facilitating the release of Cyt c from the mitochondria upstream of caspase activation. At the same time, an active thiol reducing system is needed to prevent the oxidative inactivation of caspases, allowing the transmission of the apoptotic signal. In particular, the Trx system plays an important role in maintaining caspase-9 and -3 in a reduced, active state, enabling apoptosis. During conditions of excessive accumulation of oxidants, the capacity of the cell reducing systems is overwhelmed. This results in oxidation and inactivation of caspases and attenuation of apoptosis. However, once the stress threshold is exceeded (induction of MOMP), caspase inhibition does not prevent cell death; rather, a switch from apoptosis to necrosis occurs. In some cellular settings, a redox-dependent switch from apoptosis to necroptosis is promoted through an oxidation-mediated oligomerization and activation of MLKL. In other cell contexts, excess ROS and decreased Trx activity favor the induction of pyroptosis, possibly through the oxidation and activation of GSDMD. Ineffective thiol reducing capacity, in particular depleted GSH content, can also disrupt GPX4 activity and thereby trigger ferroptosis. Thus, on the one hand, Trx/GSH support cell survival (largely by removal of oxidants); on the other hand, in the face of elevated stress and commitment to death, these reductants promote the apoptotic response. From this perspective, the proapoptotic activity of Trx/GSH can be seen as contributing to organismal homeostasis, by promoting the safe removal of damaged cells and limiting necrosis and its sequelae. This model thus provides a basis for understanding the multifunctional roles of oxidants and thiol reducing systems in cell survival/death, and also provides a framework for further dissection of the role of redox signaling in cell death modalities and the crosstalk between them.

## 8. Concluding Remarks

Recent advances provide a better understanding as to how redox-active species (ROS/RNS/RSS) and antioxidants influence specific cell death modalities. As discussed here, oxidants and antioxidants directly interact with core components of RCD pathways, such as caspases, RIPKs, MLKL and GSDMD. Thiol redox modifications of these proteins can either positively or negatively modulate apoptosis, necroptosis or pyroptosis. It should be noted, however, that in many cases, we lack a mechanistic understanding as to how a particular thiol modification alters protein structure and function. Furthermore, in some cases (such as BAX oxidation), our knowledge is derived from studies that examined how exogenous oxidants/antioxidants affect cell death progression; hence, the physiological importance of the findings remains uncertain. Nonetheless, such observations may have pharmacological relevance.

Redox regulation emerges as a potentially important mechanism for switching between RCD modalities. As noted above, distinct RCD processes can exert different pathophysiological effects, such as promotion or suppression of inflammation, and they may play divergent roles in multiple human disorders [16]. Because redox stress and dysregulated cell death are widely implicated in immune dysfunction, cancer and various degenerative diseases, further elucidation of the relationship between the two processes represents an important goal for the coming years. Redox-based manipulation of RCD pathways has therapeutic potential for many human diseases. Realizing this potential requires the development of tools that specifically target particular redox switches and cell death circuits. Accordingly, a detailed understanding of redox regulation of RCD pathways may ultimately allow the development of novel therapeutic strategies.
